# The importance of host physical niches for the stability of gut microbiome composition

**DOI:** 10.1098/rstb.2023.0066

**Published:** 2024-05-06

**Authors:** William B. Ludington

**Affiliations:** ^1^ Department of Biosphere Sciences and Engineering, Carnegie Institution for Science, Baltimore, MD 21218, USA; ^2^ Department of Biology, Johns Hopkins University, Baltimore, MD 21218, USA

**Keywords:** microbiome, niche, gut, coevolution, *Drosophila*

## Abstract

Gut bacteria are prevalent throughout the Metazoa and form complex microbial communities associated with food breakdown, nutrient provision and disease prevention. How hosts acquire and maintain a consistent bacterial flora remains mysterious even in the best-studied animals, including humans, mice, fishes, squid, bugs, worms and flies. This essay visits the evidence that hosts have co-evolved relationships with specific bacteria and that some of these relationships are supported by specialized physical niches that select, sequester and maintain microbial symbionts. Genetics approaches could uncover the mechanisms for recruiting and maintaining the stable and consistent members of the microbiome.

This article is part of the theme issue ‘Sculpting the microbiome: how host factors determine and respond to microbial colonization’.

## 1. Microbial communities are important to the health of animals, plants and the biosphere

Microbial communities live in every habitat on the Earth and play key roles in biogeochemical nutrient cycles including the carbon and nitrogen cycles, which are critical regulators of global climate change [1]. Microbial communities in soils, for instance, provide essential nutrients for plant growth and drought resistance, which influences primary productivity and carbon dioxide sequestration from the atmosphere [2,3]. In animals, the gut microbiome is crucial for the digestion of plant material, acquisition of vitamins and resistance to disease [4,5]. These processes are important for health and fitness of animals and they influence, for instance, how much plant material a herbivore can consume, which affects the local primary productivity [5]. Microbial communities in animal guts and soils also control whether carbon dioxide or methane will be released as a result of digestion and decomposition processes [6,7]. All of these microbial contributions occur at global scales, underscoring the importance of microbial communities in climate regulation.

## 2. Specific species of bacteria are consistently found in association with the gut of the same hosts

**Figure 1.**
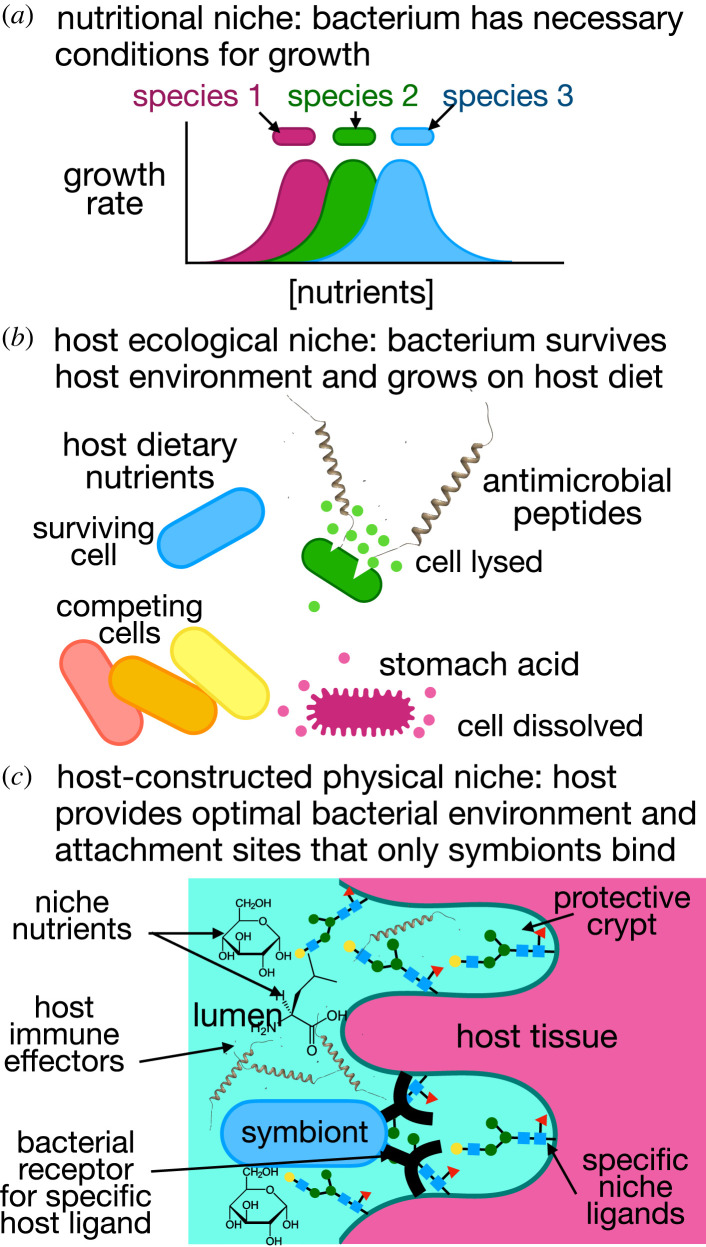
Different levels of host control for different bacterial niches in the gut. (*a*) A nutritional niche is defined by the nutrients available to a bacterium in the gut. (*b*) A host ecological niche provides dietary nutrients but also inhibits the growth of some bacteria through factors like stomach acid, bile salts, competition from other bacteria and immune effectors. (*c*) A host-constructed physical niche has the highest degree of biological sophistication. In addition to factors in the nutritional niche and host ecological niche, the physical niche provides specific sites for adhesion, protected space that limits loss of bacteria owing to peristaltic flow, and a specifically tailored nutritional and immune environment that selects the proper strains from the milieu of the intestinal flora. (Online version in colour.)

Colonization of a host gut by a bacterium is a complex process that involves the bacterium entering the host and finding a suitable environment where it can sustain a population. A bacterium requires certain nutrients, and other factors such as pH and oxygen levels are essential to growth (figure 1*a*). Inside a host, the bacterium must survive additional factors such as digestive proteases, bile salts and the immune system (figure 1*b*). The environmental filtering model of colonization postulates that the combination of microbial growth, survival of host environments, competition between microbes and random forces including chance exposures of the host to the bacteria determines colonization [8–10].

Rather than colonization being owing to chance associations alone, there might also be co-evolved fitness strategies between host and microbe that promote specific associations [11,12]. A classic example is the Hawaiian bobtailed squid, where association with a bioluminescent species of *Vibrio fischeri* is believed necessary for the squid to evade predators in the wild [13]. Both the bacterium and squid have been shown to possess numerous genes that ensure the proper association [13]. Co-phylogenetic analysis of the squid and its symbiont show a parallel evolution, where the diversification of host and symbiont show parallel phylogenies [14]. Applying a similar approach, numerous other groups have found similar co-phylogenetic diversification of host and microbe. Example hosts where this pattern occurs include sap sucking insects such as aphids [15], cicadas and sharpshooters [15,16], entomopathogenic nematodes and their bacterial symbionts [17], termites [18], stinkbugs [19–21], ants [22] and certain species of gut bacteria in primates [11,23]. While the sap sucking insects have intracellular bacteria that are vertically transmitted, the other associations are for gut bacteria, which are environmentally acquired. There are also many examples where the host and symbiont phylogenies show a lesser degree of detectable coevolution (or none at all). These include the luminous cardinalfish, *Siphamia* [24], squash bugs [25], corals [26] and sponges [27]. This tends to be true for clades that have less specific associations with their symbionts. The bacterial associations with true bugs are an example where the specificity of the insect for the bacteria is only at the level of the genus, e.g. *Burkholderia*, but competition between bacterial strains determines the strain-level specificity [20]. It is important to consider why some bacterial associations would be more or less specific. Because bugs stand to gain so much from their symbionts, it may make sense from a fitness perspective for the bug to accept any symbiont that is suitable [20]. If multiple suitable strains are present during colonization, the fittest of these is selected through symbiont–symbiont competition [20].

How bacteria are selected contributes to the co-phylogenetic patterns between host and bacterial symbiont. The posterior midgut crypts that are colonized by *Burkholderia* in bugs lie distal to the ‘constricted region’, which filters out undesirable bacteria using a combination of physical, chemical and immune mechanisms, similar to the selective mechanisms that regulate squid light organ colonization [13,20,28]. Factors such as the mechanism of symbiont acquisition (vertical versus environmental), the host diet that influences the environment for the association, and the prevalence of the bacteria in the environment can influence symbiont acquisition [29]. While a lot of emphasis has been placed on vertical transmission as the ultimate insurance of transmission, some of the best-studied hosts with symbiont specificity, such as the bobtail squid, environmentally acquire their symbionts generation after generation [30,31]. It is worth considering that if the host has a very specific need for a function that only a specific symbiont can provide, evolution can still select for mechanisms of obtaining the correct symbiont without vertical transmission [29,31]. It is in these cases without vertical transmission where we expect to find host adaptations to selectively acquire and maintain specific symbionts in host-constructed niches and to discriminate between symbiont strains to select the best ones.

## 3. Bacteria reside in physical niches in animals

Physical niches represent the utmost host control over a symbiont because they control not only which symbiont strain colonizes, but also where it colonizes, how many cells colonize and how long the symbiont cells spend in the niche before they are expelled ([13,32,33]; figure 1*c*). Physical niches hold host-selected bacterial symbionts in squid [13], fishes [34,35], bugs [28] and other insects [15]. While numerous other stochastically acquired bacteria may be present, the specific symbionts reside in privileged physical niches. These gut bacteria perform functions for the host such as the aforementioned provision of light for the squid to evade predators, breakdown of host nitrogenous waste in many insects [36] and detoxification of the diet [37,38]. For instance, *Burkholderia* in the gut of the bean bug, *Riptortus pedestris*, can break down toxic levels of the insecticide, fenitrothion, allowing the bug to survive on treated plants [38]. In addition, host physiology of the niche is influenced by the bacteria. For example, in the squid, secreted nutrients are only provisioned when the correct bacteria are present to consume those nutrients [39]. In the human stomach, crypts that house *Helicobacter pylori* regulate acid secretion, suggesting that host control of stomach pH is influenced by the correct bacterial colonizers [40]. Other physical niches include the cecum in rodents, which is thought to be a reservoir for digestive bacteria that ferment food in the colon [41]. Once thought to be a vestigial organ, the appendix in humans may serve a similar purpose to the rodent cecum [42]. Other physical niches probably exist but are yet to be discovered.

## 4. Microbiome composition is consistent within a given host species

The diversity of the microbiome is well known from 16S amplicon sequencing surveys [43]. Studies of human stool samples have routinely focused on the high inter-individual variability, and microbiome composition fluctuates wildly as people eat different meals that change the available nutrients in the gut and cause different species to bloom in abundance [44–46]. It should be noted that one of the primary data analysis techniques for analysing 16S amplicon sequencing data is principle component analysis of beta diversity (and similar techniques), which focuses on the variable characters in the data rather than the consistent ones. While weighted methods like Unifrac and Bray–Curtis do take into account differences in relative abundance, in the commonly used unweighted Unifrac method, taxa that are present in all samples are not analysed.

One of the often overlooked aspects of the human microbiome is its remarkable stability over time [47] and resiliency to perturbations including from courses of antibiotics and from diarrhoeal disease [48–50]. Furthermore, there is high consistency of the gut microbiome within an individual host species that clearly delineates different hosts based on their gut bacterial composition [51]. While factors like dietary nutrients feeding the bacteria and transmission networks of bacteria between hosts undoubtedly play a role in this consistency [52], when viewed in light of the temporal resilience of individual hosts, the evidence is also consistent with the hypothesis that host specificity is controlled through host-constructed physical niches. Such niches could guide the recolonization of the gut after diarrhoea or a course of antibiotics [48]. Given the importance of the microbiome to animal health, recolonizing with the correct bacteria would be much less risky if the host maintained reservoirs of these good bacteria in niches spaced throughout the gut, rather than starting anew each time. If the gut did start anew, then the chances of coming up with the same consortium of bacteria from those in the environment after each perturbation would be fleetingly small [53,54]. While the post-recovery microbiome certainly differs from the pre-perturbation microbiome, the degree of consistency between the two is quite high compared with a randomly sampled microbiome. This microbiome consistency is consistent with the physical niche concept.

## 5. Properties that define a niche

‘Niche’ has a range of meanings that differ between fields. In a wall, a niche is a small recess, often to hold something. When a person finds their niche in life, they have discovered a fitting lifestyle that brings contentment. In ecology, the fundamental niche is a habitat that provides the set of suitable resources and environmental conditions needed for an organism to survive, while the realized niche is the niche the organism occupies owing to interactions with other organisms [55]. In cell biology, a stem cell niche is similar to the ecological definition, but it often has a high degree of spatial constraint owing to the fact that the survival factors needed for the stem cell to live in the niche are often provided by specialized neighbouring cells [56,57]. The physical niche provided for the bacteria by the host is a protected space that also provides nutrients.

Bacterial niches in eukaryotic organisms are widespread and highly variable. There are intracellular niches, for instance in bacteriocytes of aphids and leaf hoppers, where bacterial cells are contained within the host insect cells, and these bacteria are vertically inherited from the mother [15]. There is an extensive literature on intracellular niches, and it is not discussed further here.

The non-pathogenic bacterial niches in the host gut that are the subject of this essay are extracellular, and there are many factors governing which bacteria can colonize them (figure 1). If the host provides an environment with the correct nutrients, pH and temperature for survival, then a microbe can occupy that environment in the gut, given that it can successfully compete with other gut microbes [58] (figure 1*a*). Owing to overlaps in microbial metabolisms, an ecological niche in the gut could be occupied by more than one organism [59], and a priority effect might exist, where the first organism to occupy a niche has a competitive advantage over following ones based on how it occupies the habitat [60–63]. Such ecological niches would be expected to have high variability in bacterial composition between individuals, consistent with the high degree of individuality seen in the human gut [61]. As an additional layer of host control, stomach acid, digestive proteases, bile salts and immune effectors kill susceptible bacteria (figure 1*b*). Physical niches are spatially localized sites that provide a further layer of host control (figure 1*c*). These niches are evolved sites where the host recruits and maintains specific species while excluding others (e.g. [13,32,34,64,65]). Physical niches often have a consistent set of species or strains that colonize them, and they incorporate elements of nutritional niches such as providing the correct physiological conditions. The major difference from other types of niches is the high degree of spatial specificity and selectivity that a host can exert on a physical niche. Different types of physical niches exert control on their colonizers in different ways. For example, the pouches in the posterior midgut of bean bugs seal off the symbiotic bacteria from the rest of the gut lumen, trapping them [33]. This physical barrier and the constricted region together prevent invasion by external bacteria [64]. The niche in the *Drosophila* foregut also has a pouch-like element, called the crop, but it is not sealed off from invading bacteria [32,66,67]. Instead, the bacteria physically adhere to the wall of the niche. The host could then control the colonizing species by remodeling the extracellular matrix to change the availability of the ligand that the bacteria bind. This very molecular mechanism of host control would be much more specific in terms of which bacteria can bind the niche versus being trapped in a crypt. However, these are hypotheses yet to be proved.

The exposed gut lumenal wall of the mammalian intestine is similar in that many bacteria colonize by adhering to the mucus layer secreted by the epithelial goblet cells. Lactobacilli and other mucus-adherent bacteria colonize the mucosal layer of the ileum and jejunum [68,69], and studies have found gut region-specific variation in the composition of the glycan layer in terms of its specific residues [70]. The regionality of bacterial composition of the gut correlates with the regionality of the differences in glycan composition [71]. Glycan utilization by specific gut members, such as *Bacteroidetes thetaiotaomicron's* usage of sialic acid has been biochemically and genetically investigated [72], and many of the original gut bacterial isolates, such as *Lactobacillus reuteri* and *Bifodobacterium infantis* were cultured from mucus scrapings [73]. The physical association of these bacteria with the intestinal wall makes them stable colonizers. The caecum, appendix and colonic crypts have also been recognized as niches that can store bacteria (cf. [74–76]). Differentiating stable colonizers from transient ones is important because stable colonizers have a longer-term relationship with the host and therefore are more likely to have co-evolved [73,77].

## 6. Identifying new niches

The physical niche concept is appealing because it suggests exquisite control by the host over microbiome composition and that we may be able to uncover the developmental genetic mechanisms in order to therapeutically control colonization. However, doing so in practice requires not only locating the niches but also a high degree of control over gene expression in the niche. A physical niche for bacteria was recently identified in the fruit fly *Drosophila melanogaster*, which might serve as a paradigm for the identification of other niches [32]. *Drosophila* as a host model is one of the most tractable animals for developmental genetics [78]. Individual genes can be turned on and off in specific cell types and tissues at specific times during development as controlled by the experimenter [79–82]. Moreover, *Drosophila* genetic stock centres maintain hundreds of thousands of fly lines that allow researchers to simply request the desired genetic tools in order to test the role of individual genes in specific cell types at defined times during the fly's lifespan.

The lack of a stable microbiome in laboratory-reared *Drosophila* diminished its appeal as a microbiome model [83,84]. However, stable colonizing bacteria were identified in wild *Drosophila* [32,66,67], and these strains also stably colonize laboratory flies, suggesting that the bacteria in laboratory-reared flies lost key genes for stable colonization. The stably colonizing bacteria were isolated by two different laboratories using similar approaches that clear transient bacteria from the gut while leaving stable colonizers. The assays rely on the fly's consumption of germ-free food to push unstable colonizers out of the gut. While flies typically feed on microbe-rich food, the gut clearing approaches that identified stable colonizers both used food with maintained sterility to prevent the reintroduction of bacteria [32,66,67]. Gut clearance techniques have been established in mammals with the similar goal to isolate the stable colonizers [71]. By washing the intestinal lumen and isolating bacteria from scraped mucus, researchers such as Professor Reuter isolated adherent bacteria and reduced the detection of transient bacteria [73].

To test the stability of the isolated bacteria in the fly gut, several techniques have been performed. First, bacteria were inoculated at low density and tested for their ability to colonize the gut from low initial numbers [32,66,67]. Stable colonizers reach the same population size in the gut regardless of the inoculation density [32].

To test the robustness of colonization, perturbation assays have been employed. In flies, a pulse-chase technique was developed to push out unstably adhered cells and measure the kinetics of the population turnover as a half-life [32]. This revealed a half-life on the order of weeks for a subpopulation of bacteria in the physical niche.

Pulse-chase of *H. pylori* in the stomach crypts of mice identified priority effects where the first colonizer excludes later arrivals [40]. Similar experiments making use of mice colonized by a single bacterial strain found that *Ba. thetaiotaomicron* inhabits colonic crypts with first colonizers outcompeting later arrivals [58,85]. Microscopy imaging defined the spatial locations of the colonization. Key considerations to further confirm the existence of a niche would require similar colonization of the putative niche in conventionally reared animals with a normal microbiome as well as flushing techniques to determine the stability of the niche colonizers. In flies, the specific bacteria still colonize the niche in animals with a conventional microbiome, affirming this as a true niche [32,66,67].

## 7. Conclusion

Host–microbe associations are prevalent throughout the Metazoa. While many of these associations are transient, non-specific and of little consequence to the host, many others are stable, with a high degree of specificity for both the host and microbe, and these typically have a valuable contribution to the fitness of both host and microbe [86–88]. Understanding how these specific relationships form in development and over evolutionary time is of importance to animal health and the ecological health of our planet.

The specificity of a beneficial host–microbe association is determined by both the host and the microbe, and there are various degrees of specificity with different mechanisms [11,13,15,19,28]. When the host constructs a physical niche that selectively binds with molecular specificity certain bacteria to the exclusion of others, this represents one of the highest degrees of host control [13,32]. By requiring such a high degree of specificity in a physical niche, a host can recruit specific bacteria from the highly diverse pool in the environment without falling vulnerable to pathogen invasion of the niche. Such a specific relationship provides optimal functional benefit of the symbionts to the host.

While host genetics have been elusive in the best-studied organisms such as squid and the true bugs, the discovery of a physical niche housing specific gut bacteria in *Drosophila* provides access to the best-developed toolkit for developmental genetics to study host mechanisms of symbiont specificity [32].

## Data Availability

This article has no additional data.
